# Molecular characterization of *Brucella* species detected in humans and domestic ruminants of pastoral areas in Kagera ecosystem, Tanzania

**DOI:** 10.1002/vms3.298

**Published:** 2020-06-21

**Authors:** Jean‐Bosco Ntirandekura, Victor A. Makene, Christopher J. Kasanga, Lucas E. Matemba, Sharadhuli I. Kimera, John B. Muma, Esron D. Karimuribo

**Affiliations:** ^1^ College of Veterinary Medicine and Biomedical Sciences Department of Veterinary Medicine and Public Health Sokoine University of Agriculture Morogoro Tanzania; ^2^ Département de Santé et Productions Animales Faculté d’Agronomie et de Bio‐Ingénierie Université du Burundi Bujumbura Burundi; ^3^ Department of Molecular Biology and Biotechnology The University of Dar es Salaam Dar es Salaam Tanzania; ^4^ College of Veterinary Medicine and Biomedical Sciences Department of Microbiology, Parasitology and Biotechnology Sokoine University of Agriculture Morogoro Tanzania; ^5^ The National Institute for Medical Research Dodoma Tanzania; ^6^ Department of Disease Control TheUniversity of Zambia School of Veterinary Medicine Lusaka Zambia

**Keywords:** 16S rRNA gene, *Brucella* spp., domestic ruminant, humans, Kagera region, sequencing, Tanzania

## Abstract

Brucellosis is a zoonotic disease of importance to both public health and the livestock industry. The disease is likely to be endemic in Tanzania and little is reported on molecular characterization of *Brucella* species in pastoral settings. This study aimed at characterizing *Brucella* species (targeting genus *Brucella*) infecting humans, cattle and goat in Kagera region (Ngara and Karagwe districts) using real‐time PCR, PCR amplification of 16S rRNA genes and Sanger sequencing. *Brucella* spp. were detected in 47 samples (19 sera and 28 milk) out of 125 samples (77 sera, 35 milk and 13 aborted materials) using real‐time PCR. All aborted materials (13 samples) were negative to real‐time PCR. Out of the 47 real‐time PCR positive samples (28 milk and 19 sera), 20 samples (10 milk and 10 sera) showed an expected 16S rRNA gene PCR product. Sequence analysis and blasting confirmed the presence of *Brucella* spp. in pastoral areas of Kagera region. The *Brucella* spp. from Kagera were phylogenetically grouped in two clades and three branches all closer to *B. melitensis*, *B. abortus* and *B. suis* from USA, Sudan and Iran. However, they were distinct from other species isolated also in USA, New Zealand, Germany and Egypt. This was expected based on the distance between the geographical regions from which the data (nucleotides sequences from 16S gene sequencing) for the phylogeny reconstruction were obtained. This is the first study to report *Brucella* species identified using 16S rRNA gene sequencing in East and Central Africa. A livestock vaccination program re‐inforced with a high index of *Brucella* diagnosis is needed to eradicate brucellosis in animals and minimize suffering from *Brucella* infections in humans in Tanzania.

## Graphical abstract

Authors are reporting the presence of Brucella spp. in sera (from human) also in raw milk (from cattle and goat) which could raise the suspicions of the contribution of brucellosis to abortions observed in humans and animals interacting in pastoral areas of Kagera ecosystem, in Tanzania.

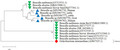

## 1 INTRODUCTION

Brucellosis is an important disease among livestock and humans in Sub‐Saharan Africa (Ducrotoy et al., 2017). In rural Africa, livestock is used as a primary source of household food as well as income from the sale of animals and their products; representing an important asset to many families (World Bank, 2013). Brucellosis occurs primarily in cattle, bison and swine, although cervids, goats, sheep and horses are also susceptible. Despite the efforts made to control the disease in many countries (Corbel, 1997), *Brucella* transmissions are persisting in domestic animals and, consequently, infections frequently occur to humans. Currently, there are 12 recognized *Brucella* species causing brucellosis (El‐Sayed & Awad, 2018) which six of them, are known to be pathogenic to humans: *B. abortus, B. canis, B. inopinata, B. melitensis, B. pinnipedialis and B. suis* (Tiller et al., 2010). Globally, *Brucella* species infecting humans and animals are often found in the human–animal ecosystem interface (Godfroid et al., 2011; Assenga, Matemba, Muller, Malakalinga, & Kazwala, 2015) where interactions are strong between humans, livestock and wildlife in the same environment. There are several studies that have reported on the presence of *B. abortus* in Uganda (Hoffman et al., 2016; Mugizi et al., 2015) and *B. abortus, B.melitensis* and *B. suis* species in Kenya (Njeru et al., 2016). These species seem to have the highest impact on domestic livestock productivity and human health in Africa (Ducrotoy et al., 2017). In Tanzania, brucellosis has mainly been documented in humans‐livestock and wildlife interfaces (Assenga et al., 2015; James, 2013; Shirima et al., 2010). However, one study reported on the isolation and characterization of *B. abortus biovar* 3 in a dairy farm in Mbeya region following abortion (Mathew et al., 2015). Consequently, there are scarce reports on molecular characterization of *Brucella* spp. which are infecting humans and livestock in pastoral settings. Various approaches have been used worldwide for identification and characterization of *Brucella* species; and for determination of origin and possible spillover to other species including humans. Actually, molecular and bioinformatics tools are giving to the knowledge an advance in understanding in single differences between species of *Brucella* evolutionary history, specificity and pathogenicity in different hosts (Vidal, Ortiz, & Olivera, 2018). Exploration of phylogenetic studies such as MLVA (Multilocus variable number of tandem repeats analysis), variable number of tandem repeats (VNTR) (Menshawy et al., 2014), single nucleotide polymorphisms (SNPs) (Wattam et al., 2014) and Multilocus sequence typing (MLST) (Shome et al., 2016) are useful for establishing relationship and grouping of *Brucella* species. Among other technics, one of the most used approach is 16S rRNA gene sequencing, which is a rapid way to confirm identity of *Brucella* species, and therefore allow implementation of needed public health responses (Gee et al., 2004; Khan et al., 2018). However, due to the variations which can be exhibited in the conserved regions of the 16S rRNA gene, it is important to design reliably of the primer for this diagnostic tool (Martinez‐Porchas, Villalpando‐Canchola, Ortiz Suarez, & Vargas‐Albores, 2017). This study aimed at molecular characterization of *Brucella* species in humans and livestock in pastoral areas of Kagera in Tanzania. Real‐time PCR was used on sera, aborted materials (from women, cattle, goats and sheep) and milk taken from cattle and goats. The 16S rRNA gene was amplified on positive samples from Real‐time PCR: sera (from women, cattle and goats) and milk (from cattle and goats) while Sanger sequencing was done using the fragment amplified from the 16S rRNA gene.

## 2 METHODOLOGY

### 2.1 Study design

**Figure 1:**
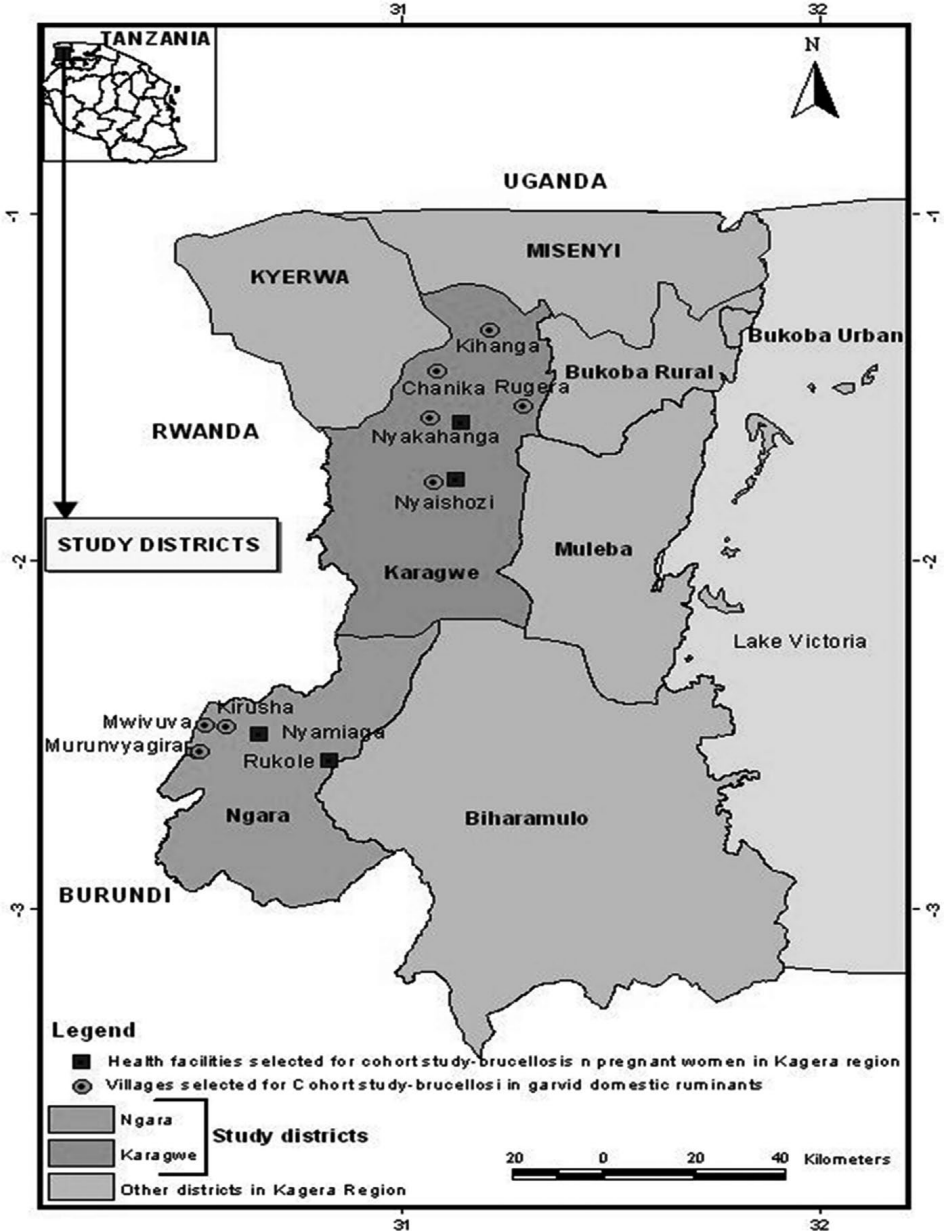
Map showing the origin of the samples used in this study (Kagera region)

The samples were collected during previous cross‐sectional and prospective cohort studies conducted in Ngara and Karagwe districts (Figure 1) in June 2017 (personal communication) and November 2017‐ April 2018 in Kagera region, Tanzania (Ntirandekura, Matemba, Kimera, Muma, & Karimuribo, 2020). Five millilitres of venous blood were firstly taken from humans going for malaria checking (July 2017) (personal communication) and from cattle, goats and sheep in the same villages. Secondly, 5ml of venous blood were taken from pregnant women attending antenatal care (November 2017‐April 2018) and gravid ruminants in the same pastoral area (Ntirandekura et al., 2020). Each blood samples reacting to the rose Bengal, C‐Elisa and FPA tests was aliquoted and kept at −20°C for molecular analysis. For the prospective study, aborted materials (from women and ruminants) and milk (from cattle, goat and sheep) were collected and stored at −20°C until the DNA extraction. During the two studies, structured questionnaires were used to complete the information on brucellosis status in the study area. Domestic ruminants were apparently healthy.

This study used sera which were positive for *Brucella* by the previous serological tests (RBPT, c‐Elisa and FPA), milk samples and aborted materials (Table 1).

**Table 1:** Description of samples used in this study

Sample/type	Species				Total
	Humans	Bovine	Goat	Sheep	
Serum (reacted to Rapid slide test, RBT, c‐Elisa and FPA tests)	28	35	12	2	77
Milk	—	23	12	—	35
Aborted materials	1	7	5	—	13
Total	29	65	29	2	125

The objective for this study was to characterize *Brucella spp*. using 16S rRNA gene sequencing. However, 16S rRNA PCR would have given too many samples for sequencing which is rather too costly. Therefore, after the DNA extraction, a real‐Time PCR (targeting genus *Brucella*) was first used to screen for *Brucella* positive samples only, then continued with a 16S rRNA PCR and sequencing.

### 2.2 DNA extraction

Genomic DNA from serum samples and aborted materials were extracted using the QIAamp DNA Mini Kit (Qiagen kit Germany) according to the manufacturer's instructions. To obtain genomic DNA from milk, the samples were centrifuged for 10 min at 10,000 × g, following which the supernatant was discarded. The GeneJET Genomic DNA Purification Kit (Thermofisher Scientific‐K0721) was used to extract DNA from the pellet, according to the manufacturer's instructions.

### 2.3 Real‐time PCR for *Brucella* spp

One hundred twenty‐five samples (Table 1) were subjected to real‐time PCR which was performed on the PikoReal machine (Thermo Fisher Scientific) for *Brucella* spp. detection. Targeting *Vdcc* gene, the forward GTGGCGATCTTGTCCG and the reverse ACGGCGATGGATTTCCG *Brucella* spp. specific primers were used (Winchell, Wolff, Tiller, Bowen, & Hoffmaster, 2010). A vaccine *Brucella* strain S19 was used as a positive control. The final reaction volume was 25 µl consisting of 2.5 µl DNA template, 1x of RealQ Plus 2x Master Mix Green (Low Rox), 10 µm of each primer, 10 µm of probe (5’ FAM‐ AAATCTTCCACCTTGCCCTTGCCATCA‐BHQ 3’) and 5.5 µl of PCR grade water. The PCR reaction started with initial heating at 50°C for 2 min, then at 95°C for 7 min, followed by 35 cycles at 95°C for 5 s and 60°C for 30 s. Data were acquired at 60°C (the extension step).

### 2.4 PCR Amplification Of 16S rRNA genes

PCR amplification of 16S rRNA genes was run on genomic DNA from 47 samples positive on real‐time PCR. Primers used for amplification were the universal 16S rRNA forward (5‐GTG‐CCA‐GCA‐GCC‐GCC‐GTA‐ATA‐C‐3) and reverse (5‐TGG‐TGT‐GAC‐GGG‐CGG‐TGT‐GTA‐CAA‐G‐3) primers according to Bricker, Ewalt, Olsen, & Jensen, (2003). The expected PCR product was 800 base pairs. DNA template (5 µl) was added to a final reaction volume of 25 µl consisting of 12.5 µl of one Taq2x master mix, 1 µl of each primer and 5.5 µl of PCR grade water. PCR reaction was run at an initial denaturation of 95 ^O^C for 5 min, followed by 40 cycles of denaturation at 95 ^O^C for 30 s; annealing at 52 ^O^C for 30 s; extension at 75 ^O^C for 90 s; and a final extension at 75 ^O^C for 5–60 min. The PCR products were visualized in a 2% agarose gel after electrophoresis at 110 volts for 60 min.

### 2.5 16S rRNA gene sequencing

Samples with a PCR amplified 16S rRNA gene were subjected to Sanger sequencing (at Sokoine University of Agriculture‐Tanzania). First PCR products were purified using GFX^TM^ PCR DNA and Gel Band Purification Kit (UK Limited Little Chalfont, Lot 16,919,854) according to the manufacturer's instructions. Then sequencing was carried out using the BigDye® Terminator v3.1 Cycle Sequencing Kit (Life Technologies). The sequencing reaction had a final volume of 10 μl. This consisted of 2 μl of 5 × sequencing buffer (mixed with 0.5 μl BigDye® Terminator v3.1) and 3 μl of appropriate sequencing primer (1.6 pmol). Sequencing primers were the forward: 5‐GTG‐CCA‐GCA‐GCC‐GCC‐GTA‐ATA‐C‐3 and reverse: 5‐TGG‐TGT‐GAC‐GGG‐CGG‐TGT‐GTA‐CAA‐G‐3 16S rRNA primers. Sequencing was done in duplicate for each primer. The sequencing conditions included incubation at 96°C for 1 min, followed by 25 cycles of denaturation at 96°C for 10 s, 50°C for 5 s and 60°C for 4 min (Bio‐Rad) on a heated lid. Following the cycle‐sequencing protocol, the reactions were cleaned up by ethanol precipitation. In this procedure, 5 µl of freshly prepared 125 mM EDTA and 60 µl of 100% ethanol were added to each reaction tube containing the sequencing products. After vortexing, the mixture was incubated in the dark for 15 min at room temperature to precipitate the extension products. As the BigDye® reagent is light‐sensitive, the precipitation was carried out in the dark. Following precipitation, the tubes were centrifuged at 13,000 × g for 30 min and the supernatant was discarded without disturbing the pellet. Subsequently, the pellets were washed with 60 µl of 70% ethanol and centrifuged at 13,000 × g for 30 min. After the supernatant had been removed, the pellets were shaded from direct light and dried in a vacuum drier until no ethanol was present. Before loading onto the ABI 3,730 DNA Analyser the samples were re‐suspended in 20 µl of HiDi Formamide (Life Technologies) and analysis was done according to the manufacturer's instructions.

### 2.6 Data analysis

Similarity searches for the 16S rRNA gene sequences obtained using Applied Biosystems 3,500 genetic analyzer (Thermo Fisher Scientific) were done using the blastn (NCBI) in GenBank databases. *Brucella spp*. gene sequences from USA and New Zealand (Gee et al., 2004), Germany (Scholz et al., 2006), Iran (Kazemi et al., 2008), Sudan (personal communication) and Egypt (Bakhiet, Mohamed, Montasser, & Abdul‐Raouf, 2013) were downloaded from GenBank (https://www.ncbi.nlm.nih.gov/) and together with Tanzanian sequences, they were used for reconstruction of phylogenetic relationship. Multiple sequence alignment (MSA) for the sequences was done using the CLUSTAL W program and phylogenetic analysis was performed using the Maximum Likelihood method and Kimura 2‐parameter model (Kimura, 1980). All analyses were done in MEGA X (Kumar *et al.,* 2018).

## 3 RESULTS

### 3.1 Real‐time PCR for *Brucella* spp

**Figure 2:**
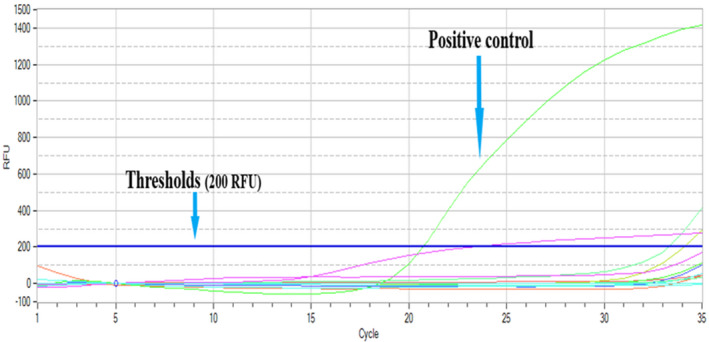
Positive control picked by the probe (with Ct value of 20.7)

**Figure 3:**
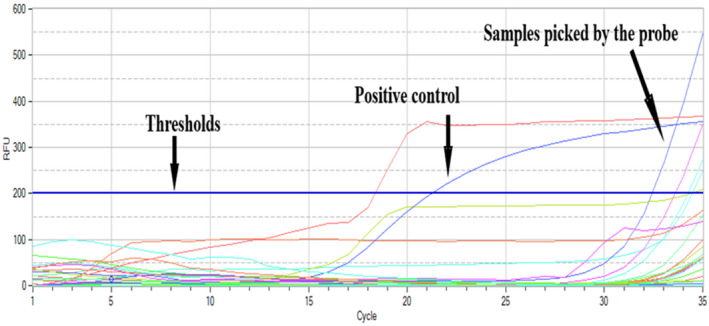
Samples picked by the probe (with Ct values ranging between 32.42 and 34.46)

Forty‐seven out of 125samples were *Brucella* positive in real‐time PCR (Table 2). The SYBR green master mix was picking well the positive control (Figure 2) in contrast to the samples under study (Figure 3). Positive control and samples were picked by the probe and were considered to be positive when numbers of cycles were enough for the fluorescence to cross the thresholds (data were acquired only in show chanel1 with thresholds of 200 RFU). All aborted materials (13 samples) were negative to real‐time PCR.

**Table 2:** Distribution of samples for molecular analysis according species

Tests	Samples type	Results according species				
		Humans	Bovine	Goat	Sheep	Total
	Serum	9 (28)	12(35)	7 (12)	0(2)	28 (77)
Real‐time PCR	Milk	—	11(23)	8(12)	—	19 (35)
	Aborted materials	0 (1)	0(7)	0(5)	—	0(13)
	Total Real time PCR	**9** (29)	**23**(65)	**15**(29)	0(2)	**47**(125)
16S rRNA amplification	Serum	3	5	2	0	10
	Milk	—	8	2	—	10
	Aborted materials	0	0	0	—	0
	Total	**3**	**13**	**4**	**0**	**20(45)**
	Serum	2	4	1	0	7
16S rRNA sequencing	Milk	—	2	1	—	3
	Aborted materials	0	0	0	—	0
	Total	**2**	**6**	**2**	**0**	**10**
Sequences used for phylogenetic reconstruction	Serum	2	0	0		2
	Milk	0	2	1		3
	Total	**2**	**2**	**1**		**5**

Bold values are explaining the total number of samples used per each test and each specie (human, bovine, caprine ans ovine).

It shows a strong positive reaction indicating abundance of target nucleic acid in the positive control used (A vaccine *Brucella* strain S19).

It shows positive reaction indicating moderate amount of target nucleic acid in our samples.

### 3.2 PCR amplification of 16S rRNA genes

**Figure 4:**
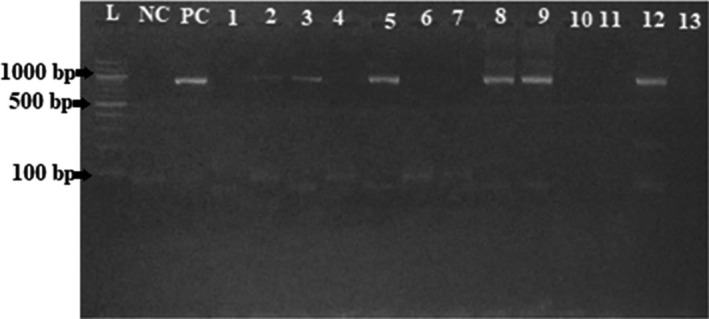
PCR amplification of 16S rRNA gene in sera from cattle

**Figure 5:**
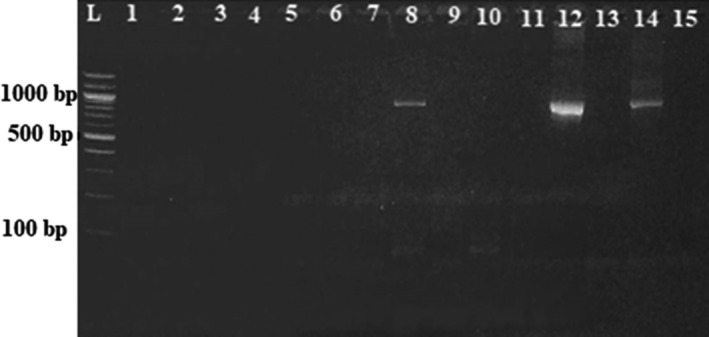
PCR amplification of 16S rRNA gene in sera from humans

The DNA fragment of *Brucella* species amplified from 16S rRNA gene is of different size according to the primers used during the amplification. In this study, we used primers (according to Bricker et al., 2003) targeting a band size of 800 base pairs. An expected 800 bp PCR product was amplified in 20 out of 47 (Table 2) real‐time PCR positive samples. Of the positive samples, five were sera from cattle (Figure 4), three sera from human (Figure 5), two sera from goats, eight milk from cow and two were milk from goats.

(L: A 100‐bp DNA ladder, NC: Negative Control; PC: Positive Control. Numbers 3, 5, 8, 9 and 12: Samples with 800 bp amplification of 16S rRNA gene. Numbers 1, 2, 4, 6, 7, 10, 11 and 13: Samples without amplification of 16S rRNA gene).

(L: A 100‐bp DNA ladder; Numbers 8, 12 and 14: Samples with 800 bp amplification of 16S rRNA gene. Numbers 1, 2, 3, 4, 5, 6, 7, 9, 10, 11, 13 and 15: Samples without amplification of 16S rRNA gene).

### 3.3 16S rRNA gene sequencing and phylogeny reconstruction

**Figure 6:**
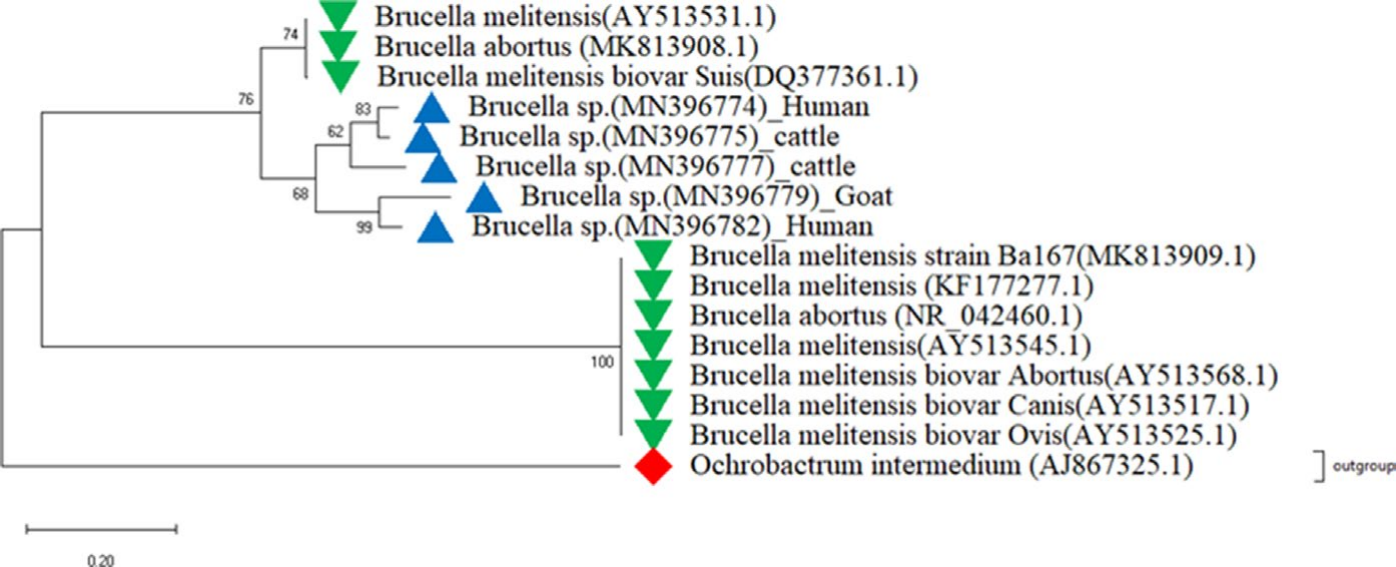
Evolutionary analysis of 16S rRNA gene sequences of samples from Kagera region in relation with other Brucella spp. by Maximum Likelihood method. 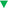 *Brucella spp* downoaloded from GenBank. 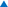 *Brucella spp* from Kagera Tanzania: MN396774: serum from aborted women. MN396782: serum from a human complaining with malaria‐like symptoms. MN396775, MN396777: milk from cattle. MN39679: milk from aborted goat. 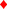 *Ochrobactrum intermedium* used in this analysis as an outgroup organism.

Sanger sequencing was successful for 10 out of the 20 samples (Table 2) with an expected PCR product size by 16S rRNA primers. The sequences were identified as belonging to *Brucella* spp. following similarity search by blastn (sequence identity of 85%). In addition, each sequence on blastn search showed same similarity with different *Brucella* spp. (*B. abortus, B. melitensis and B. suis*) deposited in Genbank considering the percent identity, the query cover and the E value. On cleaning, five sequences were used for multiple sequence alignment (MSA) and phylogeny reconstruction. The sequences were deposed in GenBank with accession numbers: MN396774, MN396775, MN396777, MN39679 and MN396782. Phylogenetic analysis of the 16S rRNA gene sequences (Figure 6) indicated that *Brucella* spp. in Kagera fall in clade closer from *B. melitensis, B. abortus* and *B. suis* reported in United States, Sudan and Iran. However, they fall also in clade away from *Brucella* spp. reported in USA, Germany, Iran and Egypt. The tree was well rooted by *Ochrobactrum intermedium* (AJ867325.1) deposited in GenBank by Lebhun *et al*. (2000) from Germany.

The evolutionary history was inferred using the Maximum Likelihood method and the Kimura 2‐parameter model (Kimura, 1980). Initial tree(s) for the heuristic search were obtained by applying the Neighbour‐Joining method to a matrix of pairwise distances estimated using the Maximum Composite Likelihood (MCL) approach. The tree is drawn to scale, with branch lengths measured in the number of substitutions per site. This analysis involved 16 nucleotide sequences. All positions with less than 95% site coverage were eliminated and there were a total of 377 positions in the final dataset.

## 4 DISCUSSION

For identifying and characterizing *Brucella* spp. in humans and domestic ruminants in Kagera region, we are reporting results of real‐time PCR, PCR amplification of 16S rRNA genes and Sanger sequencing for phylogeny reconstruction. Previously to this study, sera were pre‐screened using serological methods (Table 1), but culture of all samples (sera, milk and aborted materials) could have been used as a standard diagnostic method for isolation of *Brucella* species. However, this method is exigent, hazardous and time consuming (Bounaadja et al., 2009). Moreover the requirements in given medium for blood culture (level of CO_2_, anticoagulants, inoculum) are determining factors which could make long and fastidious process for *Brucella* isolation (Ruiz, Lorente, Perez, Simarro, & Martinez‐Campos, 1997).

This study used real‐time PCR with specific primers for *Brucella* spp. as a screening tool for *Brucella* positive samples, before embarking on 16S rRNA gene sequencing. This is because, the target region for sequencing is highly conserved among bacteria and the primers used were universal bacterial primers available to us at the time of the study. Real time PCR narrowed down our samples to 47 and has indicated the presence of *Brucella spp*. in pastoral areas of Tanzania as reported previously (Assenga et al., 2015; Mathew et al., 2015; Kassuku, 2017).

We detected *Brucella* species in cow milk, which is similar to previous reports in Egypt (Wareth, Melzer, Elschner, Neubauer, & Roesler, 2014), in Uganda (Hoffman et al., 2016) and in Tanzania (Assenga et al., 2015; Mathew et al., 2015). This calls for public health attention since some residents in the study area drink unboiled milk as it was reported previously (Ntirandekura, Matemba, Ngowi, Kimera, & Karimuribo, 2018a). We are also reporting the presence of *Brucella* in goat milk. This is in contrast to a previous study in Katavi region which could not detect any *Brucella* spp. in milk from goat (Assenga et al., 2015). However, *Brucella abortus* was detected in serum from goats in Morogoro region (Kassuku, 2017). Even though people from the study area do not consume goat milk, brucellosis in goats still poses a risk of spill over to cattle and humans in a livestock pastoral setting.

Brucellosis is reported to be associated with abortions in humans and animals (Khan, Mah, & Memish, 2001; Kurdoglu, Cetin, Kurdoglu, & Akdeniz, 2015; Mathew, 2017; Megersa et al., 2011; Muma, Godfroid, Samui, & Skjerve, 2007; Nigro et al., 2011). This study detected *Brucella* in sera from abortive woman, cow and goat. Although there was a failure to detect *Brucella* from a limited sample size of aborted materials, the detection of *Brucella* from sera of aborted individuals could raise the same suspicions regarding the contribution of brucellosis to reproductive failure in this pastoral area as reported earlier (Ntirandekura et al., 2020). The failure to detect *Brucella* species in aborted materials could have been associated to the transport medium used (liquid nitrogen), long time for conservation of samples before analysis (9 months) and the low DNA concentration among other factors. However, other factors associated with reproductive failures need to be ruled out for a causal relationship between brucellosis and abortions to hold (Ntirandekura, Matemba, Ngowi, et al., 2018).

NtirandekurReal‐time PCR positive samples were used for PCR amplification of 16S rRNA genes to get an 800 bp fragment as reported earlier (Gee et al., 2004; Khan et al., 2018). Nevertheless, a fraction of these samples showed an expected product by conventional PCR and this could be due to several optimization failures for conventional PCR assay ( Martinez‐Porchas et al., 2017). The same factors which could have affected the conventional PCR might have also affected the sequencing reaction, since a fraction of samples were successfully sequenced. Despite these limitations, we were able to get five sequences from 16S rRNA sequencing which were used in the identity search and phylogenetic reconstruction. On blastn search, each sequence was similar with different species of *Brucella* spp. deposited in Genbank, which indicated that they shared homology from a common ancestry and similar structure as stated by Pearson (2013). In addition, study sequences exhibited level of sequence similarity (with E values, percent identity and query cover) equally to *B. abortus, B. melitensis and B. suis*. It is known that the 12 recognized *Brucella* species have a genetic similarity although they differ according to their host predilection (Corbel, 2006). Hence, for phylogeny reconstruction we retrieved 16S rRNA gene sequences for *Brucella* species from GenBank. These sequences belonged to *Brucella* spp. reported from USA, Germany, Iran, Sudan and Egypt. We could not find 16S rRNA gene sequences for *Brucella* spp. isolated from East and central African regions in the DNA databases. Studies that identified *Brucella* in the region by sequencing targeted other genes (Hoffman et al., 2016; Mathew, 2017; Mugizi et al., 2015). To the best of our knowledge, this is the first study to report *Brucella* species identified by 16S rRNA gene sequencing in the region. *Brucella* spp. reported in Kagera were grouped in two clades and three branches, indicating genetic heterogeneity among the species circulating in the same region. However, all clades with Tanzanian sequences connected from clade with sequences of *B. melitensis, B. abortus and B. suis* from USA, Sudan and Iran, although there is not likely an epidemiological linkage between them.

## 5 CONCLUSION

This study showed that *Brucella* spp. are circulating in humans and their livestock in Kagera ecosystem. *Brucella* species were detected in raw milk of cow and goat, which could be a possible route of transmission to humans. In addition, the presence of *Brucella* in sera from abortive woman, cow and goat, raises the suspicion of the contribution of brucellosis to reproductive failures in the study area; despite the limited sample size. Real‐time PCR narrows down the number of samples to be sequenced and hence save cost in a limited resource setting like ours. *Brucella* spp. detected in Kagera ecosystem are phylogenetically closer to *B. melitensis, B. abortus and B. suis* reported from USA, Sudan and Iran. However they were distant from other *Brucella* spp. reported from USA, Germany, New Zealand and Egypt. There is a need to conduct more epidemiologic studies using these advanced molecular tools and contribute to the body of knowledge on the genetic and phylogenetic characteristics of this *Brucella* spp. in Kagera.

## 6 ETHICAL CONSIDERATION

This study was approved by institutional review boards of Sokoine University of Agriculture and the Medical Research Coordinating Committee of the National Institute for Medical Research (ref: NIMR/HQ/R.8a/Vol.IX/2456).

## CONFLICT OF INTEREST

The authors declare that there is no conflict of interest for this study.

## AUTHOR CONTRIBUTIONS

JBN and VAM conceived and designed the study. NJB analyzed the samples. JBN VAM and JBM analyzed the data. All authors (JBN, VAM, LEM, SIK, CJK, JBM and EDK) revised and approved the final version of this manuscript.

## Supporting information

Supplementary Material
