# Supplementary material for: Molecular characterization of Brucella species detected in humans and domestic ruminants of pastoral areas in Kagera ecosystem, Tanzania
Source: Vet Med Sci. 2020 Jun 21;6(4):711–9. doi: 10.1002/vms3.298 (PMC7738721; doi:10.1002/vms3.298)
Supplement: Supplementary file 1 — Supplementary Material [file VMS3-6-711-s001.docx]

**DNA concentration of samples used in this study**

| Sample ID | Nucleic Acid Conc. | Unit | A260 | A280 | 260/280 | 260/230 | Sample Type |
| --- | --- | --- | --- | --- | --- | --- | --- |
| SERA1 | -0.5 | ng/µl | -0.011 | -0.009 | 1.21 | -0.05 | DNA |
| SERA2 | 2.3 | ng/µl | 0.047 | 0.001 | 68.27 | 0 | DNA |
| SERA3 | 0.4 | ng/µl | 0.008 | 0.009 | 0.84 | 0.01 | DNA |
| SERA4 | 2.7 | ng/µl | 0.055 | 0.018 | 3 | 0.02 | DNA |
| SERA5 | 40.6 | ng/µl | 0.813 | 0.355 | 2.29 | 0.13 | DNA |
| SERA6 | 1.4 | ng/µl | 0.029 | -0.012 | -2.47 | 0 | DNA |
| SERA7 | 32 | ng/µl | 0.64 | 0.304 | 2.11 | 0.79 | DNA |
| SERA8 | 318.1 | ng/µl | 6.362 | 5.315 | 1.2 | 2.51 | DNA |
| SERA9 | 1.8 | ng/µl | 0.036 | 0.028 | 1.29 | 0.01 | DNA |
| SERA10 | 1.2 | ng/µl | 0.023 | 0.011 | 2.11 | 0.06 | DNA |
| SERA11 | 3.1 | ng/µl | 0.061 | 0.017 | 3.52 | 0.02 | DNA |
| SERA12 | 0.8 | ng/µl | 0.017 | 0.012 | 1.38 | 0.04 | DNA |
| SERA13 | 1.7 | ng/µl | 0.034 | 0.018 | 1.9 | 0.07 | DNA |
| SERA14 | -0.3 | ng/µl | -0.007 | -0.017 | 0.41 | 0 | DNA |
| SERA15 | 0.5 | ng/µl | 0.01 | -0.01 | -0.96 | 0 | DNA |
| SERA16 | -0.6 | ng/µl | -0.011 | -0.028 | 0.4 | 0 | DNA |
| SERA17 | 1.2 | ng/µl | 0.023 | 0.002 | 9.72 | 0.01 | DNA |
| SERA18 | -0.6 | ng/µl | -0.012 | -0.015 | 0.82 | 0 | DNA |
| SERA19 | 1.5 | ng/µl | 0.03 | 0.008 | 3.6 | 0 | DNA |
| SERA20 | 0.3 | ng/µl | 0.007 | -0.006 | -1.11 | 0 | DNA |
| SERA21 | 1.6 | ng/µl | 0.032 | 0.019 | 1.71 | 0.01 | DNA |
| SERA22 | 1.5 | ng/µl | 0.03 | 0.009 | 3.37 | 0.01 | DNA |
| SERA23 | 9.4 | ng/µl | 0.189 | 0.097 | 1.94 | 0.03 | DNA |
| SERA24 | 72.4 | ng/µl | 1.447 | 0.867 | 1.67 | 6.29 | DNA |
| SERA25 | 77.8 | ng/µl | 1.556 | 0.998 | 1.56 | 2.66 | DNA |
| SERA26 | -0.2 | ng/µl | -0.003 | -0.032 | 0.1 | 0.07 | DNA |
| SERA27 | 0.9 | ng/µl | 0.018 | -0.017 | -1.06 | -0.2 | DNA |
| SERA28 | 1.9 | ng/µl | 0.037 | 0.019 | 1.95 | -0.39 | DNA |
| SERA29 | 2.6 | ng/µl | 0.053 | 0.023 | 2.25 | -0.61 | DNA |
| SERA30 | 2.3 | ng/µl | 0.046 | 0.019 | 2.48 | -0.45 | DNA |
| SERA31 | 2.2 | ng/µl | 0.044 | 0.026 | 1.67 | -2.71 | DNA |
| SERA32 | 2.2 | ng/µl | 0.044 | 0.015 | 2.89 | -0.36 | DNA |
| SERA33 | 1.7 | ng/µl | 0.034 | -0.004 | -8.73 | -3.73 | DNA |
| SERA34 | 1.7 | ng/µl | 0.035 | 0.012 | 2.81 | -0.31 | DNA |
| SERA35 | 1.6 | ng/µl | 0.031 | -0.005 | -6.66 | 0.78 | DNA |
| SERA36 | 2.1 | ng/µl | 0.041 | 0.008 | 4.84 | -0.24 | DNA |
| SERA37 | 1.4 | ng/µl | 0.028 | -0.011 | -2.56 | -0.45 | DNA |
| SERA38 | 1.6 | ng/µl | 0.033 | -0.003 | -12.04 | 1.42 | DNA |
| SERA39 | 3 | ng/µl | 0.061 | 0.048 | 1.27 | 0.85 | DNA |
| SERA40 | 33.3 | ng/µl | 0.666 | 0.168 | 3.96 | 0.47 | DNA |
| SERA41 | 2.1 | ng/µl | 0.042 | 0.011 | 3.86 | -0.35 | DNA |
| SERA42 | 1.1 | ng/µl | 0.022 | -0.033 | -0.65 | -1.41 | DNA |
| SERA43 | 2.3 | ng/µl | 0.046 | 0.019 | 2.46 | -0.38 | DNA |
| SERA44 | 7.8 | ng/µl | 0.156 | 0.094 | 1.66 | 0.62 | DNA |
| SERA45 | 2.9 | ng/µl | 0.058 | 0.016 | 3.62 | -0.4 | DNA |
| SERA46 | 12.4 | ng/µl | 0.248 | 0.112 | 2.2 | -6.43 | DNA |
| SERA47 | 3.1 | ng/µl | 0.061 | 0.015 | 4.1 | -0.39 | DNA |
| SERA48 | 2.3 | ng/µl | 0.045 | 0.018 | 2.56 | -0.31 | DNA |
| SERA49 | 3.1 | ng/µl | 0.062 | 0.019 | 3.21 | -0.47 | DNA |
| SERA50 | 0.5 | ng/µl | 0.011 | -0.002 | -4.93 | -0.15 | DNA |
| SERA51 | 3.1 | ng/µl | 0.061 | 0.052 | 1.19 | -1.49 | DNA |
| SERA52 | 1.8 | ng/µl | 0.036 | 0.002 | 17.36 | -0.39 | DNA |
| SERA53 | 2.1 | ng/µl | 0.041 | 0.014 | 2.91 | -0.51 | DNA |
| SERA54 | 1.1 | ng/µl | 0.022 | -0.005 | -4.03 | -0.24 | DNA |
| SERA55 | -0.5 | ng/µl | -0.01 | -0.023 | 0.43 | 0.08 | DNA |
| SERA56 | 0.5 | ng/µl | 0.01 | -0.013 | -0.75 | -0.08 | DNA |
| SERA57 | 0.4 | ng/µl | 0.007 | -0.004 | -1.76 | -0.1 | DNA |
| SERA58 | 0.8 | ng/µl | 0.017 | -0.017 | -1 | 0.06 | DNA |
| SERA59 | 0.1 | ng/µl | 0.002 | -0.017 | -0.09 | -0.01 | DNA |
| SERA60 | -0.2 | ng/µl | -0.003 | -0.014 | 0.23 | 0.02 | DNA |
| SERA61 | 0.6 | ng/µl | 0.013 | -0.009 | -1.46 | -0.12 | DNA |
| SERA62 | -0.5 | ng/µl | -0.01 | -0.022 | 0.45 | 0.05 | DNA |
| SERA63 | 1 | ng/µl | 0.021 | -0.001 | -23.66 | -0.16 | DNA |
| SERA64 | 9.3 | ng/µl | 0.186 | 0.267 | 0.7 | 0.13 | DNA |
| SERA65 | 0 | ng/µl | 0 | -0.018 | 0.02 | 0 | DNA |
| SERA66 | 0.6 | ng/µl | 0.011 | -0.003 | -3.37 | -0.15 | DNA |
| SERA67 | -0.1 | ng/µl | -0.001 | -0.018 | 0.06 | 0.01 | DNA |
| SERA68 | -0.2 | ng/µl | -0.004 | -0.015 | 0.26 | 0.02 | DNA |
| SERA69 | 0.1 | ng/µl | 0.001 | -0.003 | -0.43 | -0.01 | DNA |
| SERA70 | -0.2 | ng/µl | -0.005 | -0.016 | 0.3 | 0.03 | DNA |
| SERA71 | 0 | ng/µl | 0.001 | -0.019 | -0.05 | -0.01 | DNA |
| SERA72 | 0 | ng/µl | 0 | -0.019 | -0.02 | 0 | DNA |
| SERA73 | 0.6 | ng/µl | 0.011 | 0 | -28.57 | -0.09 | DNA |
| SERA74 | -0.2 | ng/µl | -0.003 | -0.018 | 0.18 | 0.02 | DNA |
| SERA75 | 0.1 | ng/µl | 0.002 | -0.016 | -0.1 | -0.01 | DNA |
| SERA76 | -0.2 | ng/µl | -0.005 | -0.021 | 0.23 | 0.04 | DNA |
| SERA77 | 0 | ng/µl | 0.001 | -0.016 | -0.04 | 0 | DNA |
| ABORTED MATERIAL1 | 0 | ng/µl | -0.001 | -0.011 | 0.08 | 0 | DNA |
| ABORTED MATERIAL2 | -1 | ng/µl | -0.019 | -0.037 | 0.52 | 0.12 | DNA |
| ABORTED MATERIAL3 | 0.6 | ng/µl | 0.011 | 0 | -82.73 | -0.14 | DNA |
| ABORTED MATERIAL4 | 0.7 | ng/µl | 0.014 | 0.004 | 3.54 | -0.15 | DNA |
| ABORTED MATERIAL5 | 0.1 | ng/µl | 0.001 | -0.017 | -0.07 | -0.01 | DNA |
| ABORTED MATERIAL6 | 0.2 | ng/µl | 0.005 | -0.009 | -0.48 | -0.03 | DNA |
| ABORTED MATERIAL7 | 0.1 | ng/µl | 0.002 | -0.019 | -0.11 | -0.01 | DNA |
| ABORTED MATERIAL8 | 0.2 | ng/µl | 0.004 | -0.012 | -0.29 | -0.02 | DNA |
| ABORTED MATERIAL9 | 0.2 | ng/µl | 0.004 | -0.013 | -0.28 | -0.02 | DNA |
| ABORTED MATERIAL10 | -2.2 | ng/µl | -0.043 | -0.064 | 0.67 | 0.24 | DNA |
| ABORTED MATERIAL11 | -0.1 | ng/µl | -0.001 | -0.013 | 0.09 | 0.01 | DNA |
| ABORTED MATERIAL12 | -0.2 | ng/µl | -0.004 | -0.024 | 0.18 | 0.03 | DNA |
| ABORTED MATERIAL13 | 0.3 | ng/µl | 0.006 | -0.001 | -4.13 | -0.04 | DNA |
| MILK SAMPLE1 | 0 | ng/µl | 0 | -0.007 | 0.03 | 0 | DNA |
| MILK SAMPLE2 | 0.2 | ng/µl | 0.003 | -0.01 | -0.31 | -0.02 | DNA |
| MILK SAMPLE3 | 0.2 | ng/µl | 0.005 | -0.012 | -0.38 | -0.04 | DNA |
| MILK SAMPLE4 | 0.2 | ng/µl | 0.003 | -0.019 | -0.16 | -0.02 | DNA |
| MILK SAMPLE5 | 0.1 | ng/µl | 0.002 | -0.015 | -0.13 | -0.01 | DNA |
| MILK SAMPLE6 | 0.3 | ng/µl | 0.005 | -0.012 | -0.43 | -0.04 | DNA |
| MILK SAMPLE7 | -0.3 | ng/µl | -0.006 | -0.019 | 0.32 | 0.04 | DNA |
| MILK SAMPLE8 | 0 | ng/µl | 0 | -0.018 | 0 | 0 | DNA |
| MILK SAMPLE9 | -0.4 | ng/µl | -0.008 | -0.024 | 0.32 | 0.04 | DNA |
| MILK SAMPLE10 | 0 | ng/µl | 0 | -0.013 | 0.03 | 0 | DNA |
| MILK SAMPLE11 | -0.4 | ng/µl | -0.009 | -0.032 | 0.27 | 0.05 | DNA |
| MILK SAMPLE12 | 95.7 | ng/µl | 1.915 | 1.618 | 1.18 | 0.23 | DNA |
| MILK SAMPLE13 | 60.5 | ng/µl | 1.21 | 1.074 | 1.13 | 0.29 | DNA |
| MILK SAMPLE14 | 65.4 | ng/µl | 1.309 | 1.179 | 1.11 | 0.42 | DNA |
| MILK SAMPLE15 | 55.1 | ng/µl | 1.102 | 0.994 | 1.11 | 0.85 | DNA |
| MILK SAMPLE16 | 85.3 | ng/µl | 1.707 | 1.558 | 1.1 | 0.53 | DNA |
| MILK SAMPLE17 | 115.2 | ng/µl | 2.304 | 2.134 | 1.08 | 1.18 | DNA |
| MILK SAMPLE18 | 75.3 | ng/µl | 1.506 | 1.331 | 1.13 | 0.67 | DNA |
| MILK SAMPLE19 | 111.8 | ng/µl | 2.236 | 1.849 | 1.21 | 0.91 | DNA |
| MILK SAMPLE20 | 380.3 | ng/µl | 7.606 | 6.204 | 1.23 | 0.2 | DNA |
| MILK SAMPLE21 | 64.7 | ng/µl | 1.293 | 1.187 | 1.09 | 0.53 | DNA |
| MILK SAMPLE22 | 40.9 | ng/µl | 0.817 | 0.69 | 1.18 | 0.4 | DNA |
| MILK SAMPLE23 | 32.6 | ng/µl | 0.651 | 0.555 | 1.17 | 0.22 | DNA |
| MILK SAMPLE24 | 52.5 | ng/µl | 1.051 | 0.878 | 1.2 | 0.19 | DNA |
| MILK SAMPLE25 | 53.6 | ng/µl | 1.072 | 0.967 | 1.11 | 0.2 | DNA |
| MILK SAMPLE26 | 71.2 | ng/µl | 1.424 | 1.236 | 1.15 | 0.45 | DNA |
| MILK SAMPLE27 | 71.1 | ng/µl | 1.421 | 1.233 | 1.15 | 0.62 | DNA |
| MILK SAMPLE28 | 91.6 | ng/µl | 1.833 | 1.585 | 1.16 | 0.89 | DNA |
| MILK SAMPLE29 | 76.5 | ng/µl | 1.53 | 1.315 | 1.16 | 0.78 | DNA |
| MILK SAMPLE30 | 95.6 | ng/µl | 1.912 | 1.643 | 1.16 | 0.98 | DNA |
| MILK SAMPLE31 | -0.3 | ng/µl | -0.007 | -0.017 | 0.41 | 0 | DNA |
| MILK SAMPLE32 | 2.1 | ng/µl | 0.041 | 0.008 | 4.84 | -0.24 | DNA |
| MILK SAMPLE33 | 0.2 | ng/µl | 0.003 | -0.019 | -0.16 | -0.02 | DNA |
| MILK SAMPLE34 | 0 | ng/µl | 0 | -0.013 | 0.03 | 0 | DNA |
| MILK SAMPLE35 | 65.4 | ng/µl | 1.309 | 1.179 | 1.11 | 0.42 | DNA |
